# Identification of candidate genes and development of KASP markers for soybean shade-tolerance using GWAS

**DOI:** 10.3389/fpls.2024.1479536

**Published:** 2024-09-27

**Authors:** Qianru Jia, Shengyan Hu, Xihuan Li, Libin Wei, Qiong Wang, Wei Zhang, Hongmei Zhang, Xiaoqing Liu, Xin Chen, Xuejun Wang, Huatao Chen

**Affiliations:** ^1^ Institute of Industrial Crops, Jiangsu Academy of Agricultural Sciences, Nanjing, China; ^2^ Jiangsu Yanjiang Institute of Agricultural Sciences, Nantong, China; ^3^ Zhongshan Biological Breeding Laboratory (ZSBBL), Nanjing, China

**Keywords:** soybean, shade tolerance, shade tolerance coefficient, GWAS, KASP

## Abstract

Shade has a direct impact on photosynthesis and production of plants. Exposure to shade significantly reduces crops yields. Identifying shade-tolerant genomic loci and soybean varieties is crucial for improving soybean yields. In this study, we applied a shade treatment (30% light reduction) to a natural soybean population consisting of 264 accessions, and measured several traits, including the first pod height, plant height, pod number per plant, grain weight per plant, branch number, and main stem node number. Additionally, we performed GWAS on these six traits with and without shade treatment, as well as on the shade tolerance coefficients (STCs) of the six traits. As a result, we identified five shade-tolerance varieties, 733 SNPs and four candidate genes over two years. Furthermore, we developed four kompetitive allele-specific PCR (KASP) makers for the STC of S18_1766721, S09_48870909, S19_49517336, S18_3429732. This study provides valuable genetic resources for breeding soybean shade tolerance and offers new insights into the theoretical research on soybean shade tolerance.

## Introduction

Weak or low light conditions reduce the capacity of photosynthesis, which can ultimately lead to plant starvation and cause a series of disruptions in the physiological and biochemical metabolic processes throughout the plant’s entire life cycle. These disruptions include leaf curling and thinning, loss of greenery, premature leaf senescence, reduced branching, slow growth, decreased resistance, and lower plant yield and biomass (Li et al., 2012; Fankhauser and Batschauer, 2016; Li et al., 2023b; Martinez-Garcia and Rodriguez-Concepcion, 2023).

Soybean is a photophilic crop with a high demand for sunlight. However, to increase cultivation area and yield, soybeans are often interplanted with maize, sorghum, sunflower or fruit trees (Echarte et al., 2011; Ghosh et al., 2009; Yang et al., 2015; Su et al., 2023a). Under intercropping or high-density planting conditions, soybeans experience shade stress, which negatively impacts their yield and quality (Gong et al., 2015; Wu et al., 2017; Su et al., 2023a). Research has shown that weak light conditions can reduce the photosynthetic rate and chlorophyll a/chlorophyll b ration in soybean leaves, leading to a decline in photosynthetic capacity (Su et al., 2023a). Using gene/allele sequence markers (GASM-RTM-GWAS), Su et al. identified 140 genes or alleles associated with the shade-tolerance index (STI), 146 with relative pith cell length (RCL), and nine with both (Su et al., 2023b). Through transcriptome and metabolome sequence analysis of the shade-tolerant soybean ‘Nanxiadou 25’ under natural and 50% light conditions, 36 differentially expressed genes and 12 potential candidate genes related to shade tolerance were identified, including ATP phosphoribosyl transferase, phosphocholine phosphatase, AUXIN-RESPONSIVE PROTEIN, PURPLE ACID PHOSPHATASE (Jiang et al., 2023). Nandou 12 has demonstrated stronger shade resistance and a quicker recovery compared to Jiuyuehuang (shade-intolerant) during light recovery, due to its higher photosynthetic rate and smaller decrease in soluble sugar and protein content (Wang et al., 2023). Li et al. identified 29 up-regulated and 412 down-regulated proteins in soybeans seedlings exposed to 2-hour shade stress compared to those under white light. They also found that shade stress significantly impacted carbohydrate metabolic processes, especially cell wall polysaccharide biosynthetic pathways (Li et al., 2019b).

Key genes related to various agronomic traits that influence soybean shade tolerance or adaptability to high-density planting have also been identified. *PH13*, which encodes a WD40 protein and was identified through GWAS. The deletion of both the PH13 and its paralogue PHP can prevent shade-induced excessive stem elongation and enable high-density planting (Qin et al., 2023). The RIN1 (reduced internode 1) interacts with ELONGATED HYPOCOTYL 5 (HY5), STF1 and STF2 to regulate gibberellin metabolism, which controls internode length. Mutations of *RIN1* result in shorter internodes and can enhance yield in high-density planting conditions (Li et al., 2023a).

Notably, previous studies have predominantly focused on shade tolerance, which falls short of addressing the full spectrum production needs. In our study, we treated a natural population of 264 soybean accessions with a 30% reduction in light to assess their response to shade. We used the shade tolerance coefficient (STC) as the evaluation metric. Through genome-wide association study (GWAS), we identified SNPs and candidate genes associated with shade tolerance. We developed KASP markers for S18_1766721, S09_48870909, S19_49517336, S18_3429732, which have been successfully applied. This research provides new insights into the development of shade-tolerant soybean germplasm and offers valuable resources for cultivation strategies.

## Materials and methods

### Materials

A natural population consisting of 264 Chinese soybean accessions, including 212 improved varieties and 52 landraces, was utilized in this study. Genome-wide association study of the landrace panel and the cultivated panel was conducted with 2,597,425 SNPs. The particular information has been presented in our previous research (Zhang et al., 2021).

### Shade treatment and shade-tolerance evaluation

The shading stress was simulated using shade nets that reduced light by 30%, and the results were compared to normal conditions with natural light. The study took place in Nantong (32°1’N, 120°52’E), Jiangsu Province, China. Soybean germplasms were planted in June and harvested in October of both 2022 and 2023. Each soybean germplasm material was grown in 3 rows, with 10 holes per row, and each row is filled with 20-25 plants. After harvesting, six traits (first pod height, plant height, pod number per plant, grain weight per plant, branch number and main stem node number) were measured based on the *Descriptors and Date Standard for Soybean (Glycine* spp.*)* (Qiu and Chang, 2006). The shade tolerance coefficient (STC) for each trait was used as an evaluation indicator, calculated using the following formula:


$${\text{STC}}_{ij}={\bar{y}}_{ij\left(Treat\right)}/{\bar{y}}_{ij\left(CK\right)}\times 100\%$$

In which, ${\bar{y}}_{ij\left(Treat\right)}$ and ${\bar{y}}_{ij\left(CK\right)}$ represent average observed value of genotype i (i=1, 2, 3…264) on the trait j (j=1, 2, 3) with or without shade treatment (Li et al., 2014).

Standardize the STC of each genotype for each trait using the subordinate function value (SFV) (scaled to the interval ([0,1]) by the following formulas:


$$F_{ij}=\frac{\left(STC_{ij}-\min\left(STCij\right)\right)}{\left(\max\left(STC_{ij}\right)-\min\left(STC_{ij}\right)\right)}$$

$$W_{j}=pj/\sum_{j=1}^{n}pj$$

$$D=\sum_{j=1}^{n}\left[u(X_{j})*W_{j}\right]$$

In which, min (STCij) and max (STCij) represent the j (j=1, 2, 3) trait minimum and maximum of genotype i (i=1, 2, 3… 264), respectively; *W_j_* represents the importance or weight of the j trait among all composite indicators, where *pj* is the contribution rate of the j trait for each soybean genotype. The D value is the comprehensive evaluation score of shade tolerance for each soybean genotype under shade stress conditions, obtained by assessing the comprehensive indicators. Here, Xj represents the j trait (Li et al., 2014; Du, 2023).

Based on the calculated Average Subordinate Function Value (ASFV), the data for all genotypes under the current trait are evenly divided into five categories. The grouping criteria for shade tolerance in these five categories are determined, with each genotype being classified according to its shade tolerance level. The higher the ASFV, the stronger the shade tolerance of the genotype.

### GWAS

The population resequencing data utilized in this study was previously reported in our earlier research. In brief, high-density map includes 2,597,425 single nucleotide polymorphisms (SNPs) from the landrace and cultivated accessions, with a linkage disequilibrium (LD) decay range of 120 kb (Zhang et al., 2021). For each year of the study, ten plants were selected for measurement. Genome-Wide Association Studies (GWAS) were conducted using the GAPIT package based on R software and a mix linear model (MLM) were employed.

### KASP

Genotyping was performed using three sets of primers (F1, F2, and R) specifically designed for KASP markers, as detailed in **Supplementary Table S3**. These primers were designed using the Primer-Blast tool available on the NCBI website (https://www.ncbi.nlm.nih.gov/tools/primer-blast/index.cgi?LINK_ LOC=BlastHome). Genomic DNA was extracted using the 2×CTAB method (Jia et al., 2024). PCR amplification was carried out using the KASP V4.0 2×Mastermix (JasonGen, China), following the reagent’s instructions. The amplified DNA was then analyzed using a Quantitative Real-Time PCR System (ABI Quant Studio 5).

### Quantification and statistical analysis

The software IBM SPSS 20 was used for descriptive statistics and analysis of variance (ANOVA) (IBM, Armonk, NY, USA). Correlation analyses were performed using Origin software (Origin Lab, USA). The frequency distributions of six traits for the soybean accessions in both years were calculated by Microsoft Excel 2016.

## Results

### Shade-tolerance evaluation and analysis of the six agronomic traits across the 264 soybean accessions with or without shade treatment

To evaluate shade tolerance, we cultivated a natural soybean population of 264 accessions under conditions with or without 30% shade treatment in Nantong during 2022 and 2023. Six agronomic traits (first pod height, plant height, main stem node number, pod number per plant, grain weight per plant, and branch number) were measured across the 264 accessions over two years. Overall, the average first pod height and plant height in 2022 (E1) and 2023 (E2) under shade treatment were higher than those under normal light conditions. In contrast, the average main stem node number, pod number per plant, grain weight per plant, and branch number showed varying degrees of decline (**Supplementary Table S1**). To evaluate the shade tolerance of the soybean population, STC for six traits was calculated, and descriptive statistical analysis were performed to the 264 accessions from 2022 and 2023 (**Table 1**). The STC values for six traits were defined as STC1-6, respectively. As shown, the mean STC values for these six traits in 2022 and 2023 did not exhibit significant differences (**Table 1**), with heritability (h^2^) values of 43.64%, 40.15%, 44.57%, 36.52%, 40.61% and 38.99%, respectively (**Table 1**).

**Table 1:** Descriptive statistics of STCs of six traits across 264 soybean accessions with or without shade treatment.

Trait	Year	Max	Min	Mean		SD	CV (%)	Skewness	Kurtosis	*h^2^* (%)
STC1	E1	7.00	0.36	1.74	1.76	1.04	59.43	1.817	5.085	43.64
	E2	5.19	0.59	1.77		0.77	43.65	1.278	2.68	
STC2	E1	8.54	0.76	1.92	1.91	0.80	41.38	3.215	20.50	40.15
	E2	3.61	0.23	1.90		0.63	32.96	0.417	-0.050	
STC3	E1	1.96	0.56	1.05	1.05	0.25	24.11	0.873	0.737	44.57
	E2	1.99	0.51	1.04		0.27	25.52	0.516	0.057	
STC4	E1	2.42	0.19	0.90	0.98	0.39	43.72	1.171	1.924	36.52
	E2	4.29	0.25	1.06		0.51	48.20	2.114	8.463	
STC5	E1	6.97	0.00	0.93	1.09	0.68	73.17	3.646	26.463	40.61
	E2	5.28	0.22	1.24		0.74	60.20	2.222	7.798	
STC6	E1	10.00	0.00	1.09	1.18	1.43	130.92	3.823	17.216	38.99
	E2	12.33	0.00	1.26		0.98	77.83	6.931	73.241	

STC1, STC of first pod height; STC2, STC of plant height; STC3, STC of node number on main stem; STC4, STC of pod number per plant; STC5, STC of seed weight; STC6, STC of branch number. Max, maximum; Min, minimum; SD, standard deviation; CV, coefficient of variation; *h^2^*, heritability.

**Figure 1:**
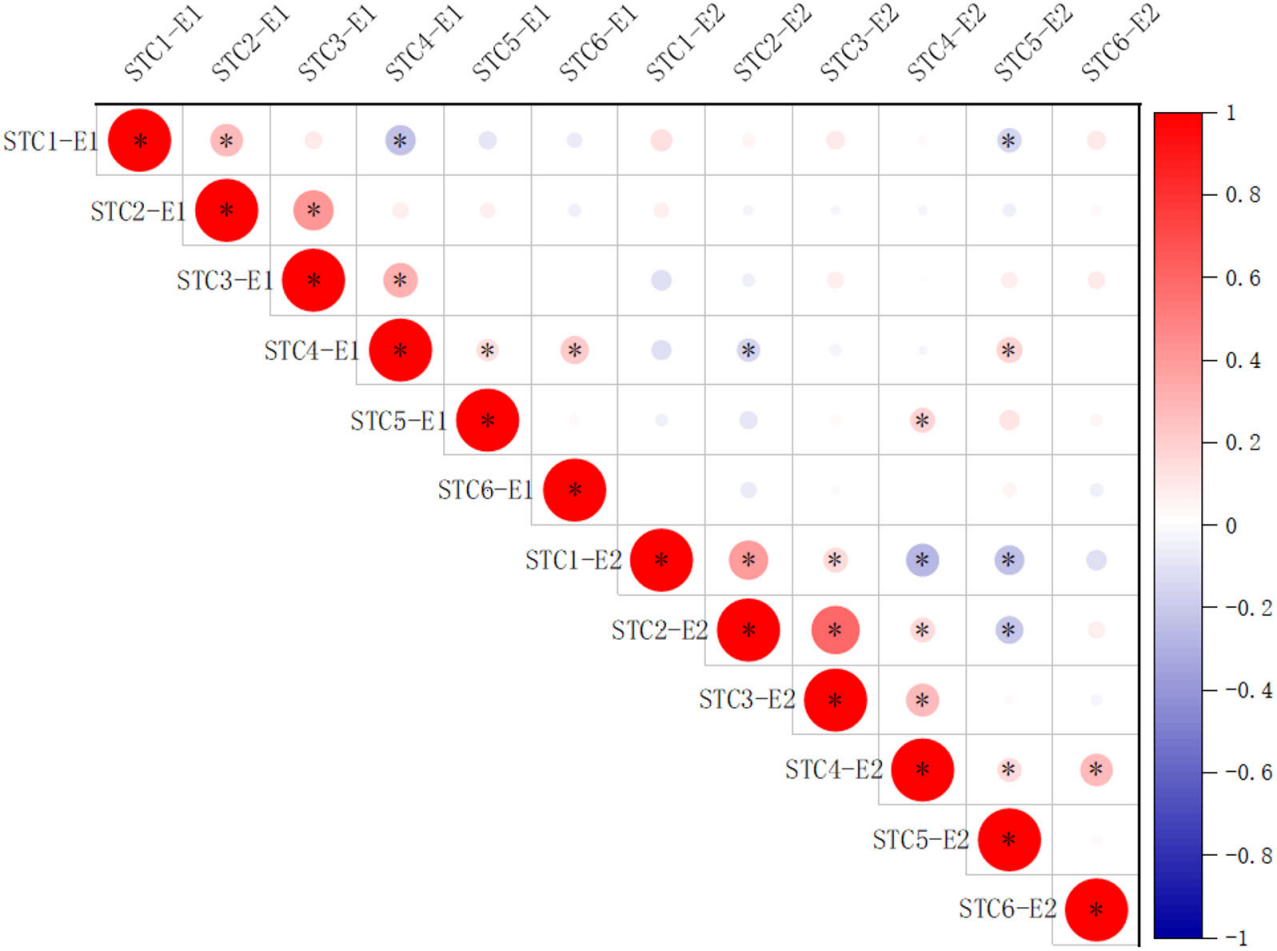
Correlation analysis among STC1, STC2, STC3, STC4, STC5, and STC6 of 2022 and 2023. E1, 2022; E2, 2023. *Represents *P*<0.05.

An analysis of variance (ANOVA) was conducted on the six traits across the 264 accessions in 2022 and 2023, revealing significant differences among genotypes, stress treatments, and different environments (**Table 2**). To explore the correlation among the six traits in 2022 and 2023 for the soybean population, a correlation analysis was conducted (**Figure 1**). The results indicated that the STC1 and STC2, STC2 and STC3, STC3 and STC4, STC4 and STC6 showed significant positive correlation in 2022 and 2023 (**Figure 1**). While, STC1 and STC4 in 2022, STC1 and STC4, STC5 in 2022, STC2 and STC5 in 2023 exhibited significant negative correlation (**Figure 1**). Between two years, only STC4 in 2022 and STC2 in 2023, STC1 in 2022 and STC5 in 2023 shown negative correlation, STC5 in 2022 and STC4 in 2023, STC4 in 2022 and STC5 in 2023 shown significant positive correlation, respectively. But other traits between two years shown weak significant correlation (**Figure 1**).

**Table 2:** Variance analysis of six traits in soybean natural population.

Trait	Variation source	Square Sum	Mean Square	F value	P value
First pod height	G	900.129	4.018	2.241	<0.01
	E	0.286	0.286	0.159	0.69
	G×E	760.974	3.397	1.895	<0.01
Plant height	G	421.892	1.883	2.582	<0.01
	E	0.509	0.509	0.698	0.404
	G×E	465.538	2.078	2.849	<0.01
Stem node number	G	69.076	0.308	3.05	<0.01
	E	0.214	0.214	2.121	0.146
	G×E	63.242	0.282	2.792	<0.01
Pod number per plant	G	161.9	0.723	1.725	<0.01
	E	0.041	0.041	0.097	0.756
	G×E	187.62	0.838	1.999	<0.01
Seed weight per plant	G	133.354	0.595	1.246	0.027
	E	7.309	7.309	15.294	<0.01
	G×E	87.727	0.392	0.82	0.954
Branch number	G	307.1	1.371	1.848	<0.01
	E	0.007	0.007	0.009	0.923
	G×E	314.363	1.403	1.892	<0.01

G, genotype; E, environment; SS, square sum; MS, mean square.

### Shade tolerance soybean germplasms

**Figure 2:**
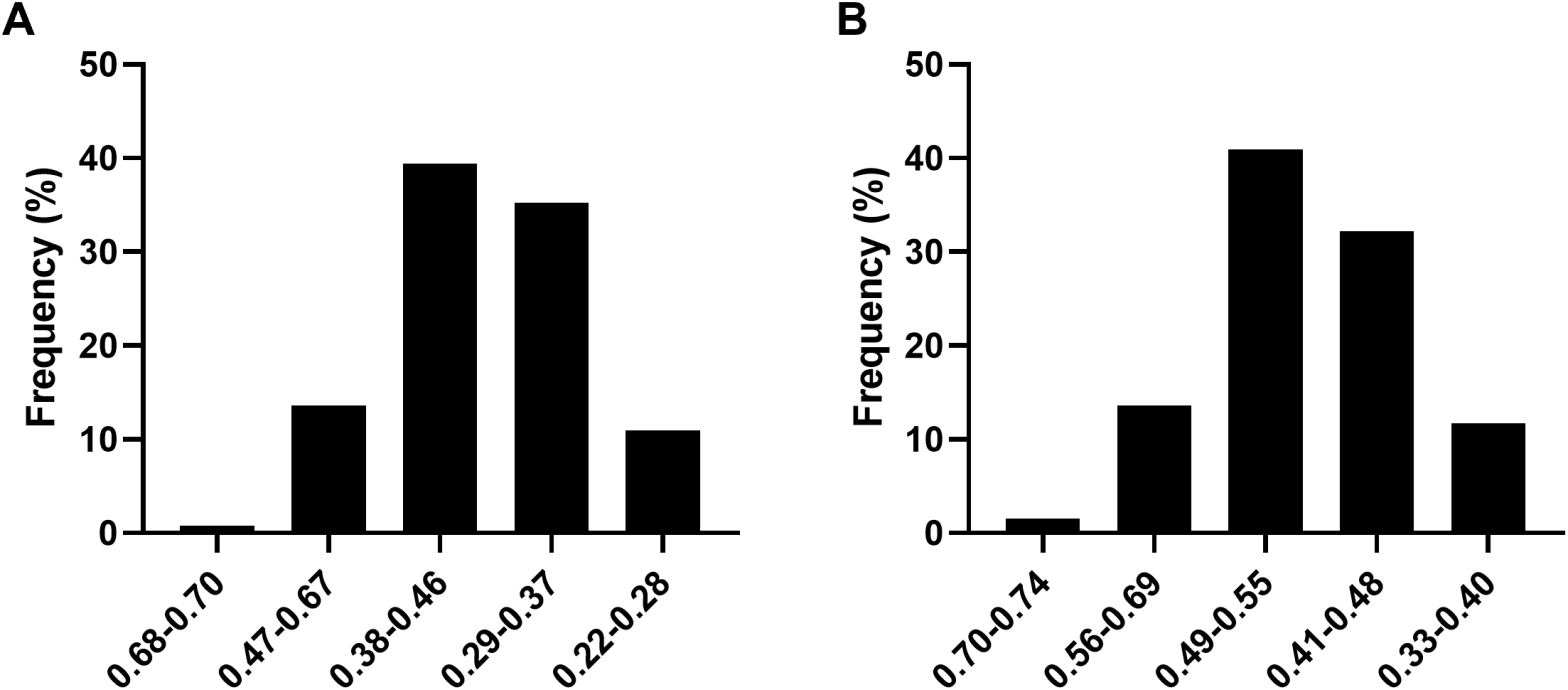
ASFV of 2022 **(A)** and 2023 **(B)**.

In 2022, the ASFV thresholds for different levels of shade tolerance were as follows: high shade tolerance was above 0.68, shade tolerance ranged from 0.47 to 0.68, moderate shade tolerance ranged from 0.38 to 0.47, shade sensitivity ranged from 0.29 to 0.38, and high shade sensitivity was below 0.29 (**Figure 2A**). In 2023, the thresholds were slightly adjusted: high shade tolerance was above 0.70, shade tolerance ranged from 0.56 to 0.70, moderate shade tolerance ranged from 0.49 to 0.56, shade sensitivity ranged from 0.41 to 0.49, and high shade sensitivity was below 0.41 (**Figure 2B**). Over the two years, moderate shade tolerant soybean germplasm was the most prevalent, comprising approximately 39% and 41% of the total population. Shade sensitive germplasm ranked second after moderate shade tolerant germplasm. High shade tolerant germplasm was relatively rare, accounting for 0.76% and 1.52% of the total soybean population in 2022 and 2023, respectively (**Figure 2A**).

In summary, this study identified a total of five high shade tolerant soybean germplasms over the two years, with NPS044 being selected in both years (**Table 3**). These high shade tolerant materials offer a valuable foundation for further research into the genetic mechanisms underlying soybean shade tolerance and serve as important experimental materials for future breeding programs aimed at enhancing shade tolerance in soybeans.

**Table 3:** Shade-tolerant soybean germplasms that were screened in 2022 and 2023.

Classification	High shade tolerance	ASFV
2022	NPS044	0.70
	NPS060	0.69
2023	NPS044	0.74
	NPS187	0.73
	NPS254	0.73
	NPS151	0.72

### GWAS for six agronomic traits and STCs across the 264 soybean accessions with or without shade treatment

**Figure 3:**
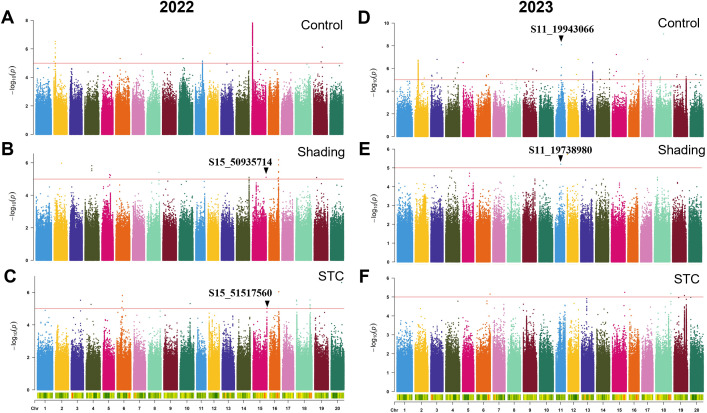
GWAS for first pod height with or without shade treatment in 2022 and 2023. **(A–C)** control, shade treatment and STC of 2022; **(D–F)** control, shade treatment and STC of 2023, respectively. Red lines represent-log10(*p*)≥5.0.

**Figure 4:**
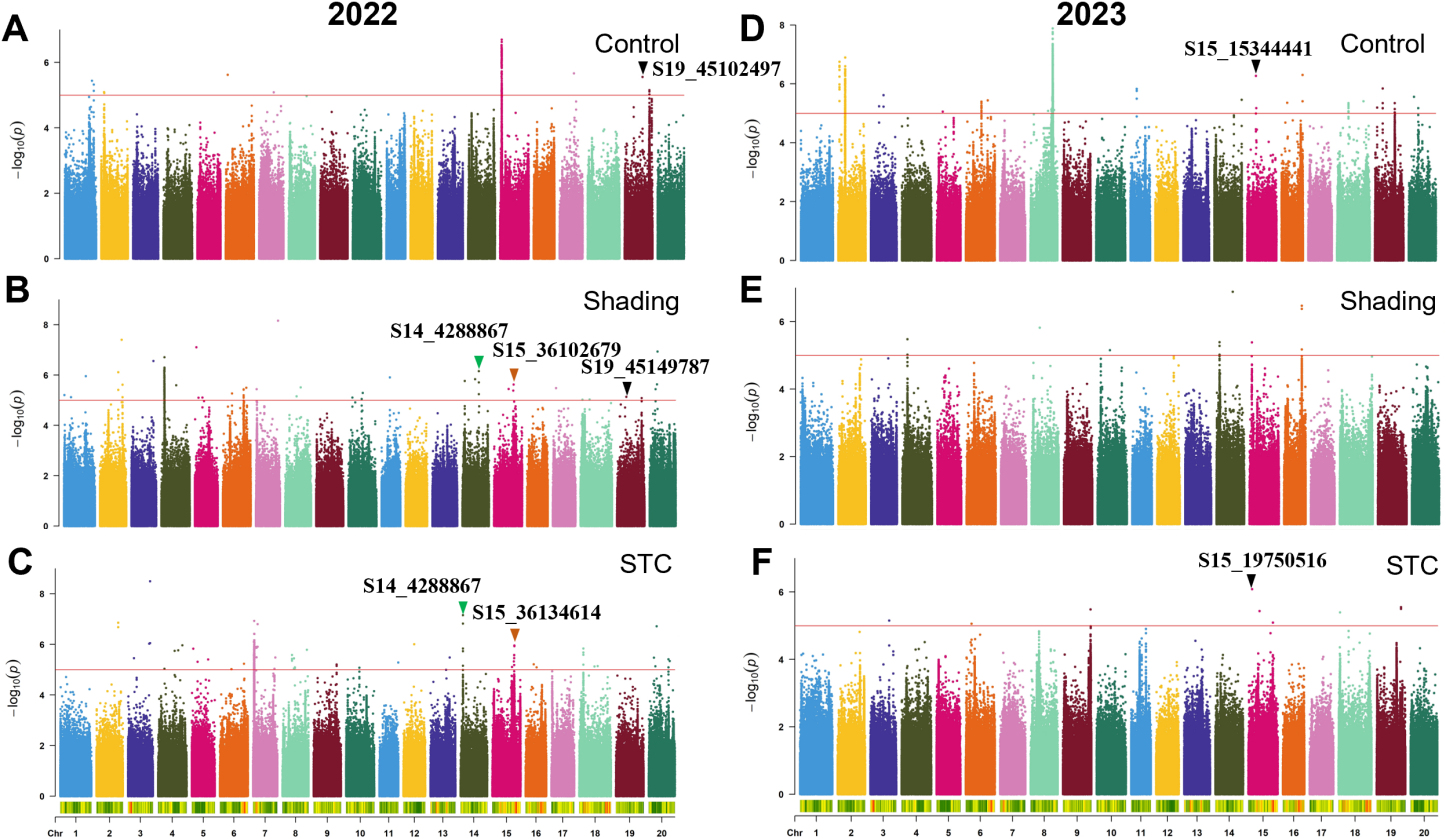
GWAS for plant height with or without shade treatment in 2022 and 2023. **(A–C)**, control, shade treatment and STC of 2022; **(D–F)**, control, shade treatment and STC of 2023, respectively. Red lines represent–log_10_(*p*)≥5.0.

**Figure 5:**
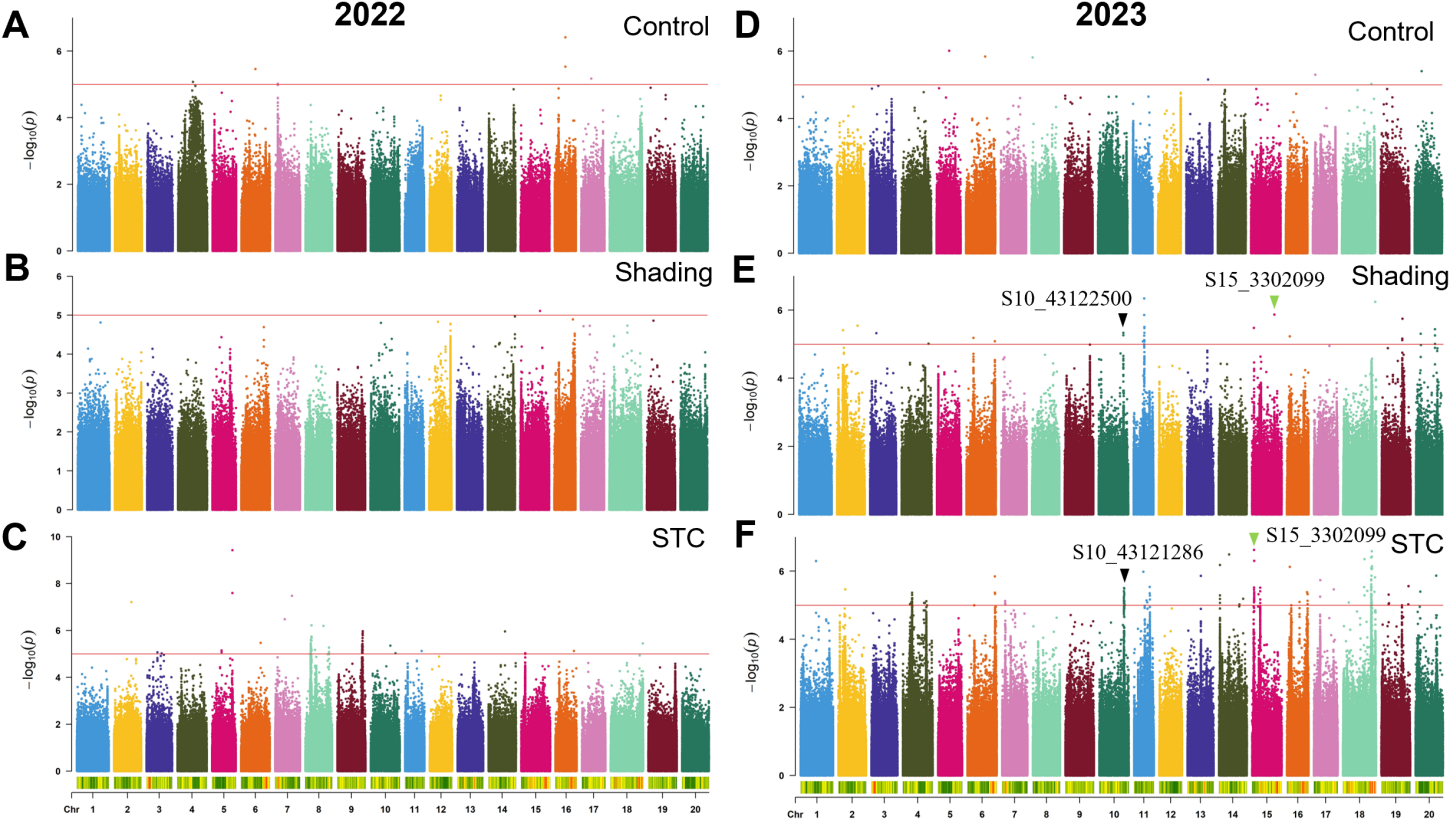
GWAS for grain weight with or without shade treatment in 2022 and 2023. **(A–C)**, control, shade treatment and STC of 2022; **(D–F)**, control, shade treatment and STC of 2023, respectively. Red lines represent–log_10_(*p*)≥5.0.

**Figure 6:**
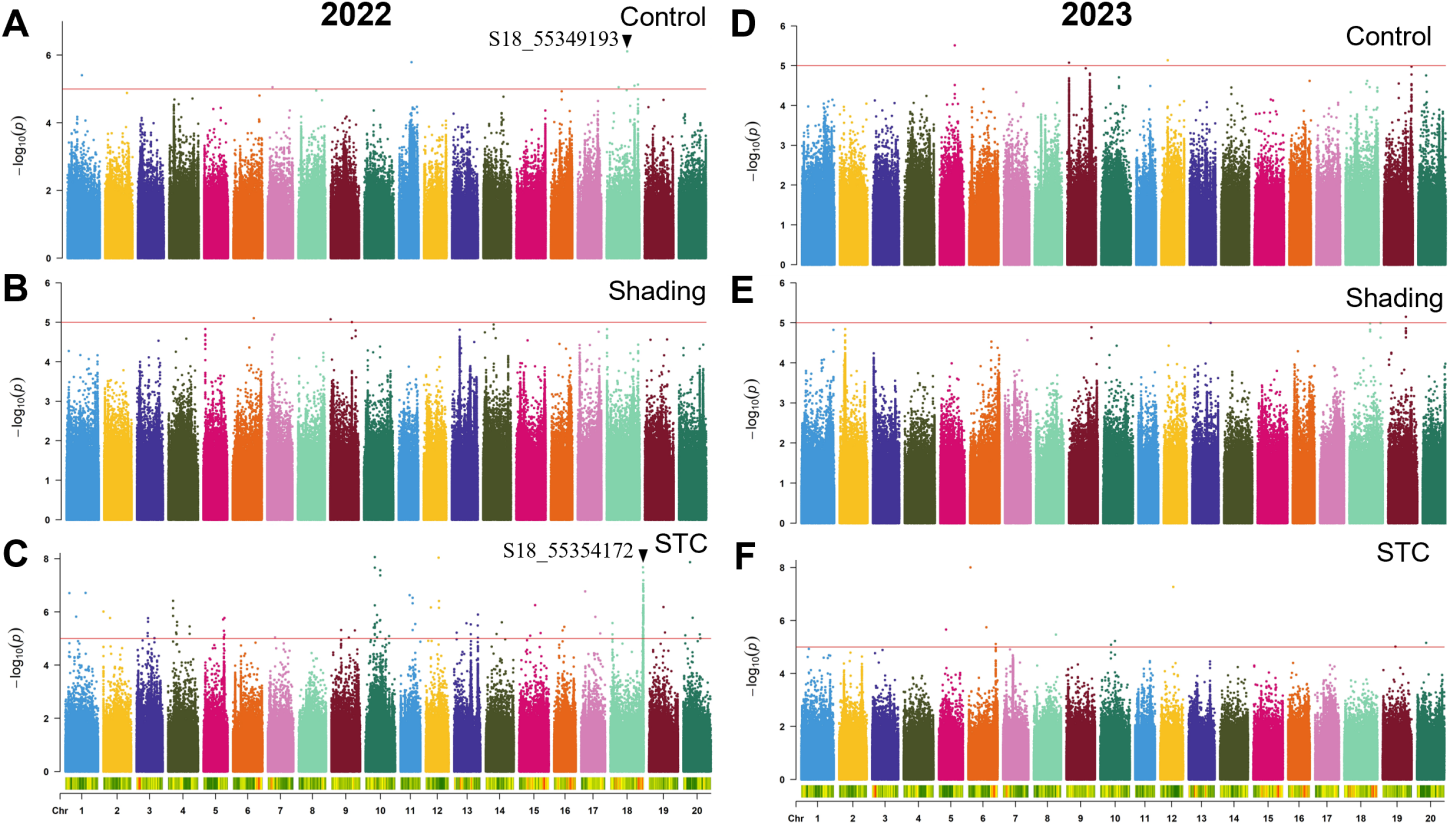
GWAS for branch number per plant with or without shade treatment in 2022 and 2023. **(A–C)**, control, shade treatment and STC of 2022; **(D–F)**, control, shade treatment and STC of 2023, respectively. Red lines represent–log_10_(*p*)≥5.0.

To pinpoint key genomic loci responsible for shade tolerance in soybeans, we conducted GWAS on six traits across 264 soybean accessions under control and shade conditions, as well as STC of the six traits for the years 2022 and 2023 (**Figures 3**–**6**; **Supplementary Figures S3**, **S4**). The resulting frequency distribution maps and density curves indicated that the phenotypic data for the six traits followed a continuous distribution. This suggests that the natural soybean population in our study harbors rich genetic variation, making it well-suited for further GWAS analyses (**Supplementary Figures S1**, **S2**).

Over the course of two years, we identified a total of 733 significant SNPs associated with STCs of six traits (**Table 4**). Specifically, for the STC of first pod height, 28 SNPs were detected. In 2022, 24 SNPs were associated with the STC of first pod height, while in 2023, 4 SNPs were identified. Notably, S11_19943066 was significant under control conditions, whereas S11_19738980 was significant under shade, with these two SNPs being approximately 204 kb apart (**Figures 3A, B**; **Table 4**). In 2023, S15_50935714 was significant under shade treatment, while S15_51517560 showed a significant correlation with the STC, with a distance of approximately 582 kb between these two SNPs (**Figures 3E, F**; **Table 4**).

**Table 4:** GWAS analysis results for six traits associated with shade tolerance.

Env.	Trait	Significant SNP Number	-log_10_(*p*)		R^2^ (%)	
		-log_10_(*p*) ≥5.0	Max	Min	Max	Min
2022	STC1	24	6.61	5.01	10.87	7.86
	STC2	29	7.15	5.08	12.56	8.42
	STC3	2	6.12	6.12	10.69	10.69
	STC4	13	6.21	5.05	10.94	8.58
	STC5	105	9.28	5.00	18.02	8.74
	STC6	227	8.48	5.01	15.87	8.58
2023	STC1	4	5.25	5.08	9.12	8.78
	STC2	9	6.08	5.06	10.18	8.20
	STC3	2	5.41	5.29	9.44	9.21
	STC4	31	6.61	5.00	12.67	9.15
	STC5	190	6.63	5.00	12.22	8.78
	STC6	97	22.62	5.00	53.76	8.77
Total		733				

In 2022, 29 SNPs were linked to the STC of plant height, while in 2023, 9 SNPs were identified. The SNP S15_15344441 showed significant association under control conditions, and S15_19750516 was significantly correlated with the STC (**Figures 4A, B**; **Table 4**). Moreover, S19_45102497 and S19_45149787 were significantly associated with plant height under control and shade conditions, respectively, with a distance of approximately 47 kb between them. The SNP S14_4288867 was significantly correlated with both plant height and STC. Additionally, S15_36102679 and S15_36134614 were significantly associated with soybean plant height under shading conditions and STC, with these two SNPs being approximately 32 kb apart (**Figures 4D–F**; **Table 4**).

For the main stem node number, two SNPs showed significant correlation with the STC. The SNPs S04_11807969 and S14_4288867 were notably correlated with both the main stem node number under shading and the STC. Additionally, 2 SNPs were significantly correlated with the STC in 2023 (**Supplementary Figure S3**; **Table 4**).

For the trait of pod number per plant, 13 SNPs were identified as significantly correlated with the STC of pod number per plant in 2022. Additionally, 31 SNPs were significantly associated with the STC (**Supplementary Figure S4**; **Table 4**).

Regarding grain weight per plant, a total of 105 SNPs in 2022 and 190 SNPs in 2023 were significantly correlated with the STC. Specifically, the SNP S15_1302099 showed a significant correlation with both single plant grain weight and the STC of soybean under shading. The SNP S10_43122500 was significantly associated with grain weight per plant under shading conditions, while S10_43121286 was significantly correlated with the STC, with these two SNPs being approximately 1 kb apart. Notably, the SNP associated with the STC for grain weight per plant had the highest explanatory power, with a -log_10_(*p*) value of 9.28 and a phenotype explanatory rate of 18.02% (**Figure 5**; **Table 4**).

A total of 324 SNPs were found to be significantly correlated with the STC of branch number. Specifically, 227 SNPs in 2022 and 97 SNPs in 2023 were significantly associated with the STC (**Table 4**). In 2023, the SNP S18_55349193 showed significant correlation with branch number under control conditions, while S18_55354172 was significantly correlated with the STC, with these two SNPs being approximately 5 kb apart (**Figure 6**).

### Development and application of KASP markers for soybean shade tolerance

**Figure 7:**
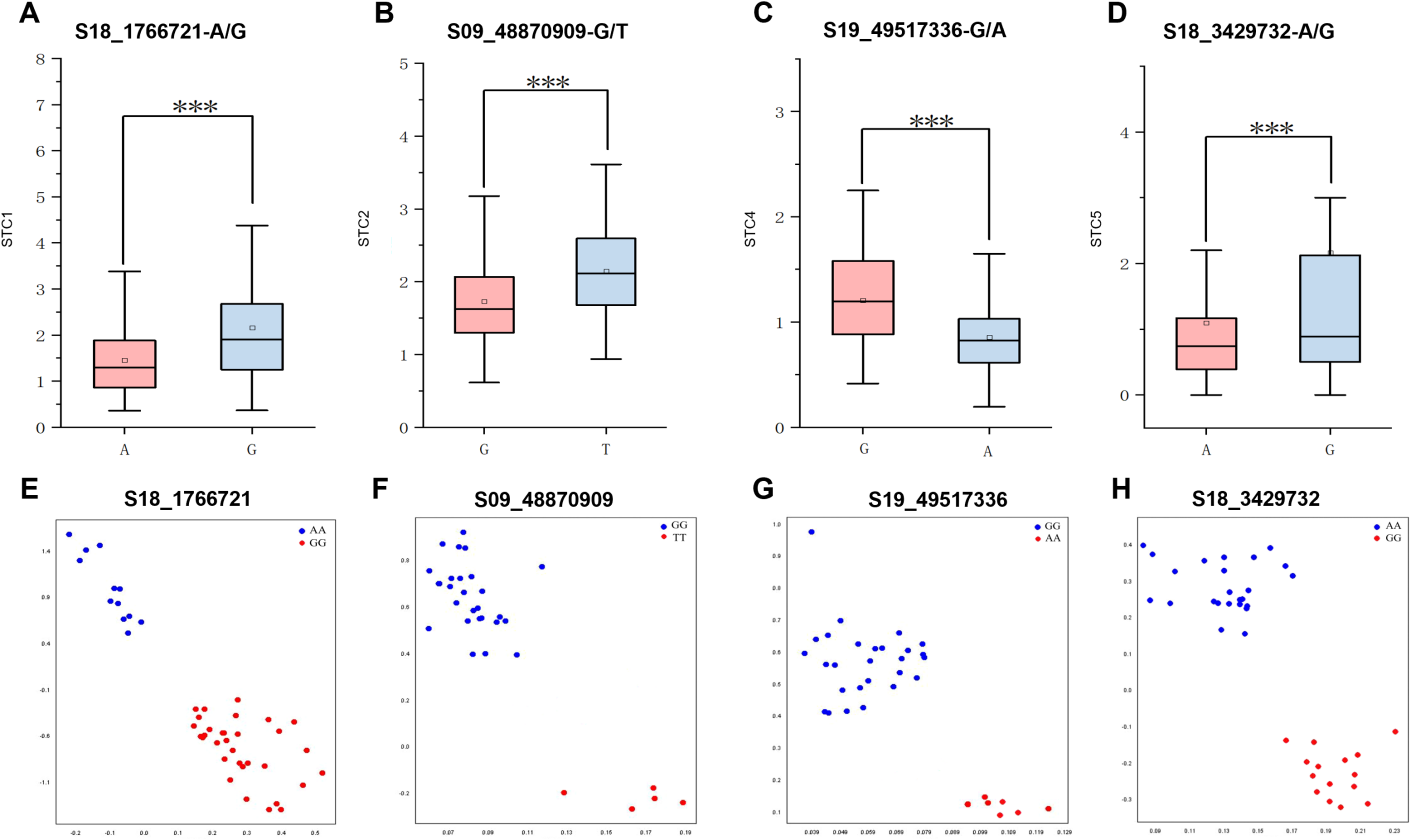
Haplotype analysis and genotyping for S18_1766721 **(A, E)**, S09_48870909 **(B, F)**, S19_49517336 **(C, G)** and S18_3429732 **(D, H)**, respectively. *** represents P<0.001.

To explore the phenotypic effects of allelic variations in significant SNPs, a haplotype analysis was conducted on the SNPs with the highest threshold detected for shade tolerance during the mature stages of 2022 and 2023. This analysis revealed a total of 4 SNPs showing significant differences between each genotype. For instance, the SNP S18_1766721 exhibited an allelic variation from A to G. The STC of first pod height was significantly higher in germplasm carrying the S18_1766721-G allele compared to those with the S18_1766721-A allele (**Figure 7A**). Another example includes the S09_48870909, which has a G/T allelic variation (**Figure 7B**). The nucleotide change at position S19_49517336 involves a substitution from G to A. Soybean with the S19_49517336-G allele exhibit a significantly higher average STC of pod number per plant compared to those with the S19_49517336-A allele (**Figure 7C**). For the S18_3429732, the allelic variation consists of A and G. Soybean germplasm with the S18-3429732-A allele has a significantly higher STC for average grain weight per plant compared to those with the S18-3429732-G allele (**Figure 7D**). The phenotypic variation explain rate of S18_1766721, S09_48870909, S19_49517336, and S18_3429732 are 8.32%, 9.01%, 8.93%, 8.73%, respectively. The favorable alleles ratio in the population of S18_1766721-G, S09_48870909-T, S19_49517336-G and S18_3429732-G were 37.9%, 34.5%, 87.5%, and 13.6%, respectively (**Table 5**). And the HST germplasms NPS044, NPS060, NPS151, NPS187 and NPS254 each contained three, three, two, four and one favorable alleles (**Table 5**). Also, we developed KASP markers for these four SNPs (**Table 6**). The designed molecular markers effectively differentiate between these two genotypes (**Figures 7E–H**).

**Table 5:** The number of favorable alleles present in the five high shade-tolerant soybean germplasms.

SNP	Ratio (%)	NPS044	NPS060	NPS151	NPS187	NPS254
S18_1766721-G/A	37.9/62.1	A	G	A	G	A
S09_48870909-T/G	34.5/65.5	T	T	G	T	G
S19_49517336- G/A	87.5/12.5	G	G	G	G	G
S18_3429732-G/A	13.6/86.4	G	A	G	G	A

Ratio represents the allele ratio in the population.

**Table 6:** Primers used for KASP.

SNP	Primer	Primer sequence (5’-3’)
S18_1766721 (STC1)	F1 F2 R	**GAAGGTGACCAAGTTCATGCT** TAAAAAAAAATGACAATTAG **A** **GAAGGTCGGAGTCAACGGATT** TAAAAAAAAATGACAATTAG **G** TGGCATCCACTCATGAAATCG
S09_48870909 (STC2)	F1 F2 R	**GAAGGTGACCAAGTTCATGCT** TCATTGATGATAGTATGGTT **G** **GAAGGTCGGAGTCAACGGATT** TCATTGATGATAGTATGGTT **T** GTGTTTCACAACTGCTGGGC
S19_49517336 (STC4)	F1 F2 R	**GAAGGTGACCAAGTTCATGCT** ATCTAATTTTAATTTACAGT **A** **GAAGGTCGGAGTCAACGGATT** ATCTAATTTTAATTTACAGT **T** ACGAATTGTGTTGGCTGTAACC
S18_3429732 (STC6)	F1 F2 R	**GAAGGTGACCAAGTTCATGCT** TGTAGAAAACGCGCTTTGTA **A** **GAAGGTCGGAGTCAACGGATT** TGTAGAAAACGCGCTTTGTA **G** TGACAACGACATATGCAAACACAA

Underlines/bold sequences in F1 indicate Field Application Manager (FAM) fluorescent junction sequence and underlines in F2 indicated Hexachlorofluorescein (HEX) fluorescent junction sequence. The bold/underline characters indicate SNPs.

### Identification candidate genes for shade tolerance based on GWAS

The LD of this population is 120 kb (Zhang et al., 2021). Therefore, we examined candidate genes within a 120 kb range upstream and downstream of SNPs significantly associated with soybean STC across six traits. Utilizing functional annotation information from the soybean genome, we identified four candidate genes significantly linked to soybean shade tolerance (**Table 7**). *Glyma.18G024000* associated with S18_1766721, encodes a trichome birefringence-like 33 protein. The gene *Glyma.09G271100* linked to S09_48870909, encodes a protein from the auxin efflux carrier family. *Glyma.19G248900*, associated with S19_49517336, encodes an ethylene response factor 1. Lastly, the gene *Glyma.18G040700*, related to S18_3429732, encodes a MYB domain protein 43.

**Table 7:** Functional annotation of candidate genes related to shade tolerance in soybean.

Trait	Gene ID	Homologs	Functional annotation
STC1	*Glyma.18G024000*	*AT2G40320*	Trichome birefringence-like 33
STC2	*Glyma.09G271100*	*AT5G01990*	Auxin efflux carrier family protein
STC4	*Glyma.19G248900*	*AT3G23240*	Ethylene response factor 1
STC6	*Glyma.18G040700*	*AT5G16600*	MYB domain protein 43

## Discussion

### Shade treatment, evaluation and shade-tolerance germplasms

In natural environments, plants are often subjected to shade tolerance. Shade tolerance is essential for soybeans, especially in intercropping or relay cropping systems. When soybeans experience shade stress, their plant height and first pod height will be elongated and the branches, number, grain weight per plant, pod number and nodes number will be reduced, which posed a huge threat to soybean production (Yang et al., 2015; Raza et al., 2020; **Table 1**).

Previous studies have demonstrated that a 15% reduction in light is considered weak shading, whereas 60% shading often leads to lodging in most varieties, indicating excessive shading. However, at 30% shading, the proportion of lodging varieties and the coefficient of phenotypic variation are sufficient to meet the requirements for shade tolerance identification (Sun et al., 2017; Zhang, 2021). Therefore, this study employed a 30% light reduction to simulate shade treatment. As a result, under 30% shading, all the six traits exhibited more pronounced phenotypic changes, and the result all present normal distribution (**Supplementary Table S1**; **Supplementary Figures S1**, **S2**).

ASFV has been widely utilized for assessing crop resistance to various stressors, including salt, drought and shade tolerance (Zhao et al., 2023). In this study, ASFV was applied to evaluate the shade tolerance of soybean (**Figure 2**). Five germplasms exhibiting high shade tolerance were identified: NPS044, NPS060, NPS151, NPS187 and NPS254. Notably, NPS044 showed consistent results across two years (**Table 3**). Chen et al. (2003) measured various parameters such as STC of biological yield during pod setting, plant height, minimum pod height, pod number per plant, grain number per plant, grain weight per plant, and 100 grain weight, and calculated the ASFV of soybean varieties. Similarly, Huang et al. (2012) employed a comprehensive STC across nine indicators, including standard pod number, standard pod weight, 100 grain weight, plot yield, plant height, main stem node number, number of ingle grain pods number per plant, single plant pod weight per plant, and standard pod length, to determine soybean shade tolerance. Li et al. (2014) developed a mathematical model for evaluating soybean shade tolerance using stepwise regression and identified seven key indicators: main stem node number, branch number, internode length, lodging resistance, pod number per plant, 100-grain weight, and grain weight per plant. Wu et al. (2015) suggest that the rapid identification and prediction of shade tolerance in soybean seedlings can be achieved by measuring leaf dry weight, stomatal conductance, plant height, and maximum fluorescence yield under dark conditions. Tang et al. (2022) used traits such as first pod height, stem node number, pod number per plant, and grain number per pod to evaluate shade tolerance. In this study, six traits - first pod height, plant height, pod number per plant, grain weight per plant, branch number and main stem node number – were measured using STC as the indicator to evaluate shade tolerance in 264 soybean accessions. These traits are reliable for identifying key loci and genes associated with shade tolerance in soybeans.

### Shade-tolerance SNPs and candidate genes associated with soybean shade tolerance

A total of 733 SNPs were identified as being associated with the STC of six traits over two years (**Figures 3** **–** **6**; **Supplementary Figures S3**, **S4**; **Table 4**). Due to the significant influence of environmental factors on these traits (**Supplementary Figure S1**), we didn’t co-locate any significant loci between two years. More environments may need to be added.

Based on GWAS, four SNPs S18_1766721, S09_48870909, S19_49517336 and S18_3429732 were selected for further study. Their phenotypic explanation rate ranged from8.32% to 9.01%, which can be used for soybean genome selection breeding. Four candidate genes associate with the four SNPs were identified. *Glyma.18G024000*, associated with S18_1766721, encodes the protein Trichome birefringence-like 33 (TBL33). Members of the TBL family, previously characterized, are localized in the Golgi apparatus and function as polysaccharide O-acetyltransferases catalyzing the O-acetylation of specific cell wall polymers (Stranne et al., 2018; Sinclair et al., 2020; Lunin et al., 2020). TBL proteins have been reported to play roles in biotic (disease, herbivore) and abiotic resistance (salt, drought and freezing) (Xiong et al., 2013; Gao et al., 2017; Sun et al., 2020). Glyma.09G271100 is an auxin efflux carrier family protein, known as PIN -like (PILS) which plays a crucial role in auxin signaling (Bogaert et al., 2022; Feraru et al., 2022; Waidmann et al., 2023). Numerous studies have demonstrated that auxin plays pivotal roles in integrating responses to abiotic stresses such as temperature, water, light and salt and in controlling downstream stress responses (Iglesias et al., 2018; Waadt et al., 2022; Xie et al., 2022; Jing et al., 2023). Organ-specific transcriptome analysis has revealed that shade induces a set of auxin-responsive genes, such as SMALL AUXIN UPREGULATED RNAs (SAURs) and AUXIN/INDOLE-3-ACETIC ACIDs (AUX/IAAs) (Nguyen et al., 2023). In the initial response to shade signals, auxin biosynthesis, transport, and sensitivity are rapidly activated, promoting cell elongation in hypocotyls and other organs (Ma and Li, 2019). *Glyma.19G248900* associated with S19_49517336, encodes an ethylene response factor 1 (ERF1). *GmERF3* has been reported to positively regulates resistance to virus, high salinity and dehydration stresses (Zhang et al., 2009; Liu et al., 2024). Ethylene is known to play a crucial role in mediating plant adaptations to environmental conditions (Waadt et al., 2022). Recent studies have shown that shade stress can induce ethylene biosynthesis, accelerating soybean senescence and hindering nitrogen remobilization (Deng et al., 2024). *ERFs* are significant in enhancing flood tolerance in rice (Xu et al., 2006), where ethylene accumulation in submerged tissues induces the expression of *ERFs* such as SNORKEL1 and SNORKEL2, which are major QTLs associated with deepwater internode elongation (Hattori et al., 2009). *Glyma.18G040700* related to S18_3429732, encodes MYB domain protein 43. This protein plays a critical role in various aspects of plant growth and development, including secondary metabolic regulation, responses to hormones and environmental factors, cell differentiation, organ morphogenesis, and cell cycle regulation (Li et al., 2019a). The homolog AtMYB43 has been reported to be involved in regulating tolerance to cadmium and freezing (Zheng et al., 2022, 2023). In summary, these four genes *Glyma.18G024000*, *Glyma.09G271100*, *Glyma.19G248900*, and *Glyma.18G040700* may be involved in soybean responses to shade tolerance.

### KASP markers for soybean shade tolerance

This study developed four KASP markers based on SNPs associated with soybean STC obtained from GWAS. These markers have been successfully used for genotyping (**Table 7**; **Figure 7**). Specifically, S18_1766721 is associated with the STC of first pod height, S09_48870909 with the STC of plant height, S19_49517336 with the STC of pod number per plant, and S18_3429732 with the STC of branch number. These markers are valuable tools for identifying shade-tolerant soybean germplasms and can enhance the efficiency and accuracy of selection in molecular marker-assisted breeding. However, KASP markers for the STC of node number and grain weight were not developed. This gap may be due to the influence of multiple factors, suggesting that further efforts and research are needed to identify effective markers for these traits.

## Data Availability

The datasets presented in this study can be found in online repositories. The names of the repository/repositories and accession number(s) can be found in the article/**Supplementary Material**.
