# Supplementary material for: Identification of candidate genes and development of KASP markers for soybean shade-tolerance using GWAS
Source: Front Plant Sci. 2024 Sep 27;15:1479536. doi: 10.3389/fpls.2024.1479536 (PMC11466877; doi:10.3389/fpls.2024.1479536)
Supplement: Supplementary file 1 [file DataSheet1.docx]

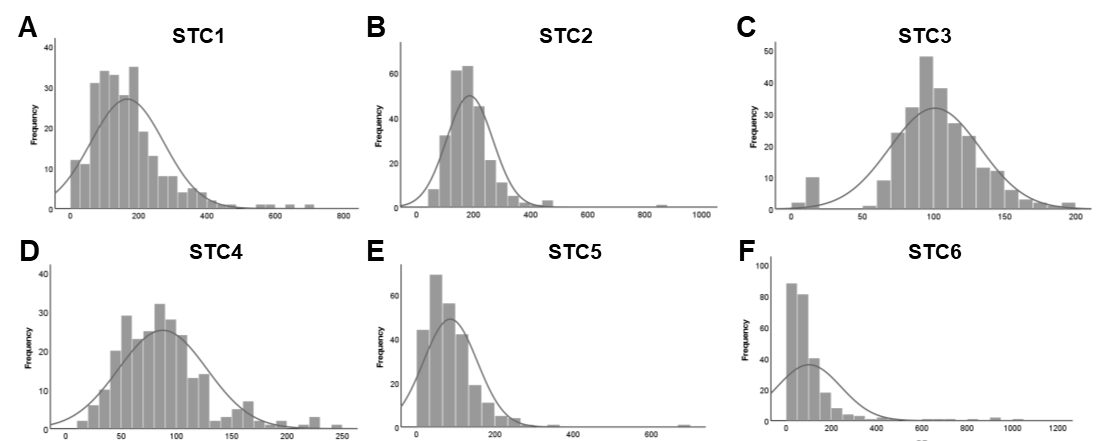


**Supplemental Figure S1** Frequency distribution of STC1, STC2, STC3, STC4, STC5 and STC6 in E1.


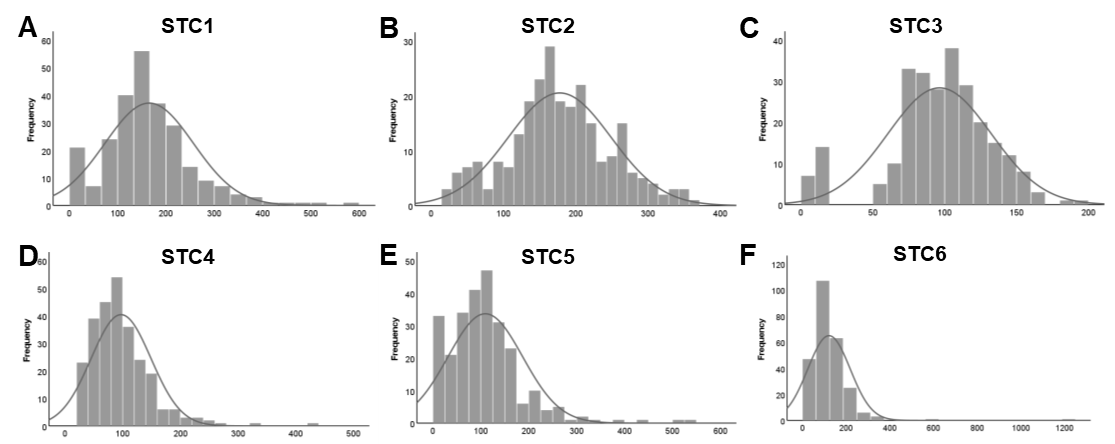


**Supplemental Figure S2** Frequency distribution of STC1, STC2, STC3, STC4, STC5 and STC6 in E2.


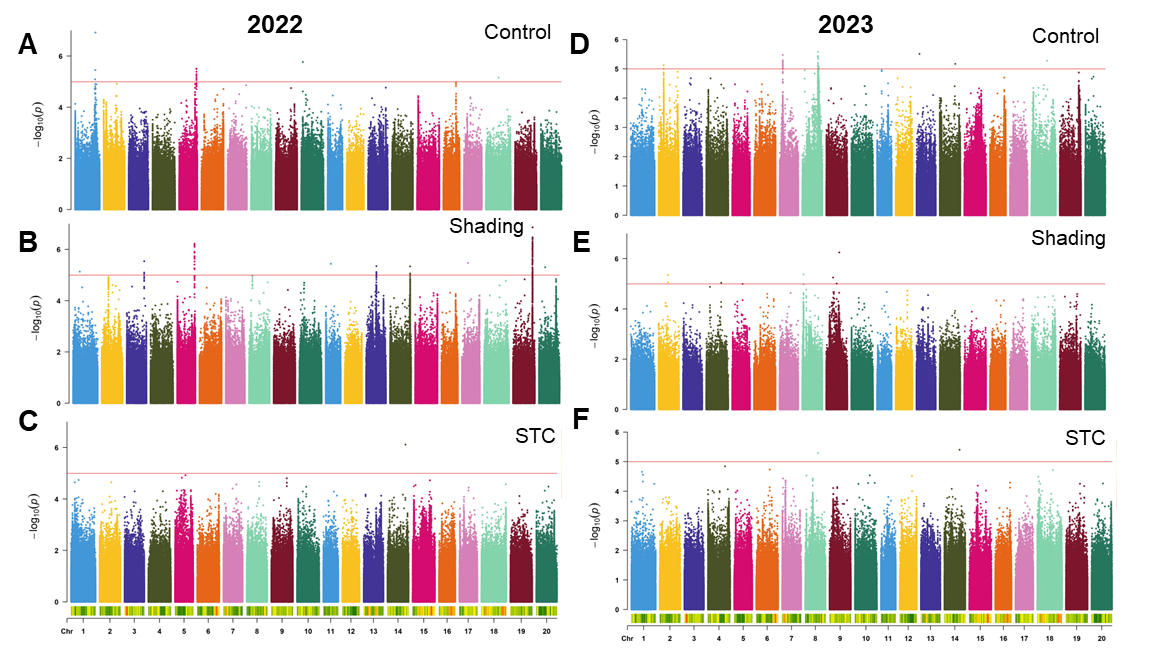


**Supplemental Figure S3** GWAS for node number per plant with or without shade treatment in 2022 and 2023. A, B and C, control, shade treatment and STC of 2022; D, E and F, control, shade treatment and STC of 2023, respectively. Red lines represent –log_10_(*p*)≥5.0.


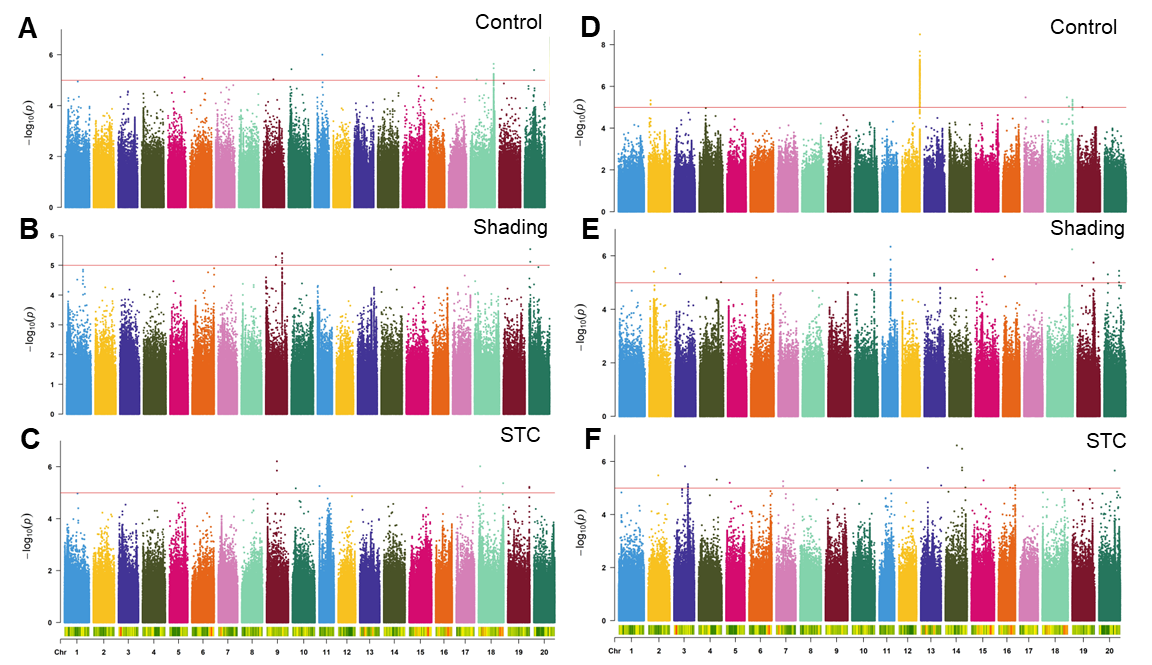


**Supplemental Figure S4** GWAS for pod number per plant with or without shade treatment in 2022 and 2023. A, B and C, control, shade treatment and STC of 2022; D, E and F, control, shade treatment and STC of 2023, respectively. Red lines represent –log_10_(*p*)≥5.0.
