# Supplementary material for: Identification of candidate genes and development of KASP markers for soybean shade-tolerance using GWAS
Source: Front Plant Sci. 2024 Sep 27;15:1479536. doi: 10.3389/fpls.2024.1479536 (PMC11466877; doi:10.3389/fpls.2024.1479536)
Supplement: Supplementary file 2 [file Table1.docx]

**Supplemental Table S1** Descriptive statistics of six traits across 264 accessions with or without shade treatment

| Trait | Year | | Treat | | Max | Min | | | Range | | | Mean | | | | SD | | CV (%) |  |
| --- | --- | --- | --- | --- | --- | --- | --- | --- | --- | --- | --- | --- | --- | --- | --- | --- | --- | --- | --- |
| First pod height (cm) | | E1 | C | 25.67 | | | 2.33 | | | 2.33~25.67 | | 11.26 | | | 5.28 | | 46.85 | | |
|  |  |  | T | 33.67 | | | 4.00 | | | 4.00~33.67 | | 16.22 | | | 6.07 | | 37.43 | | |
|  |  | E2 | C | 35.56 | | | 4.80 | | | 4.80~35.56 | | 11.75 | | | 3.99 | | 33.98 | | |
|  |  |  | T | 39.44 | | | 4.60 | | | 4.60~39.44 | | 19.22 | | | 6.30 | | 32.78 | | |
| Plant height (cm) | | E1 | C | 142.00 | | | | 19.00 | | 19.00~142.00 | 56.89 | | | | 17.92 | | 31.50 | | |
|  |  |  | T | 223.33 | | | | 33.33 | | 33.33~223.33 | 101.69 | | | | 33.78 | | 33.22 | | |
|  |  | E2 | C | 103.40 | | | | 22.50 | | 22.50~103.40 | 46.67 | | | | 13.98 | | 29.96 | | |
|  |  |  | T | 128.86 | | | | 10.40 | | 10.40~128.86 | 82.93 | | | | 16.36 | | 19.72 | | |
| Main stem node number | | E1 | C | 20.00 | | | 5.83 | | | 5.83~20.00 | 13.09 | | | 2.75 | | | 21.02 | | |
|  |  |  | T | 19.33 | | | 6.67 | | | 6.67~19.33 | 13.33 | | | 2.15 | | | 16.15 | | |
|  |  | E2 | C | 15.80 | | | 6.80 | | | 6.80~15.80 | 11.11 | | | 1.75 | | | 15.72 | | |
|  |  |  | T | 17.90 | | | 6.00 | | | 6.00~17.90 | 11.31 | | | 2.18 | | | 19.29 | | |
| Pod number/  plant | | E1 | C | 147.00 | | | 16.67 | | | 16.67~147.00 | 53.17 | | | 20.49 | | | 38.53 | | |
|  |  |  | T | 109.00 | | | 14.67 | | | 14.67~109.00 | 43.31 | | | 15.57 | | | 35.96 | | |
|  |  | E2 | C | 108.67 | | | 16.83 | | | 16.83~108.67 | 42.09 | | | 14.97 | | | 35.57 | | |
|  |  |  | T | 94.10 | | | 9.11 | | | 9.11~94.10 | 33.22 | | | 11.57 | | | 34.83 | | |
| Seed weight/  plant (g) | | E1 | C | 52.67 | | | 3.20 | | | 3.20~52.67 | 19.33 | | | 8.77 | | | 45.38 | | |
|  |  |  | T | 44.43 | | | 0.00 | | | 0.00~44.43 | 14.86 | | | 6.75 | | | 45.43 | | |
|  |  | E2 | C | 49.47 | | | 5.00 | | | 5.00~49.47 | 15.73 | | | 6.56 | | | 41.70 | | |
|  |  |  | T | 40.24 | | | 2.86 | | | 2.86~40.24 | 13.90 | | | 5.93 | | | 42.69 | | |
| Branch number | | E1 | C | 8.00 | | | 0.00 | | | 0.00~8.00 | 2.58 | | 1.26 | | | | 48.92 | | |
|  |  |  | T | 6.50 | | | 0.00 | | | 0.00~6.50 | 1.99 | | 1.26 | | | | 63.50 | | |
|  |  | E2 | C | 8.00 | | | 0.33 | | | 0.33~8.00 | 3.27 | | 1.27 | | | | 38.75 | | |
|  |  |  | T | 5.90 | | | 0.20 | | | 0.20~5.90 | 2.76 | | 1.09 | | | | 39.65 | | |

Notes: E1, 2022; E2, 2023; C, Control (non-shade treatment); T, shade treatment; Max, maximum; Min, minimum; SD, standard deviation; CV, coefficient of variation. The same as below.
